# Transparent Fiber-Reinforced Composites Based on a Thermoset Resin Using Liquid Composite Molding (LCM) Techniques

**DOI:** 10.3390/ma14206087

**Published:** 2021-10-14

**Authors:** Yavuz Caydamli, Klaus Heudorfer, Jens Take, Filip Podjaski, Peter Middendorf, Michael R. Buchmeiser

**Affiliations:** 1 Institute of Polymer Chemistry (IPOC), University of Stuttgart, Pfaffenwaldring 55, D-70569 Stuttgart, Germany; yavuz.caydamli@ipoc.uni-stuttgart.de; 2 German Institutes of Textile and Fiber Research (DITF), Körschtalstr. 26, D-73770 Denkendorf, Germany; 3 Institute of Aircraft Design (IFB), University of Stuttgart, Pfaffenwaldring 31, D-70569 Stuttgart, Germany; heudorfer@ifb.uni-stuttgart.de (K.H.); take@ifb.uni-stuttgart.de (J.T.); 4 Nanochemistry Department, Max-Planck-Institute for Solid State Research, Heisenbergstr. 1, D-70569 Stuttgart, Germany; podjaski@fkf.mpg.de

**Keywords:** transparency, glass fiber-reinforced polymer (GFRP), liquid composite molding (LCM), epoxy, thermoset, E-glass, mechanical, optical, transmittance

## Abstract

In this study, optically transparent glass fiber-reinforced polymers (tGFRPs) were produced using a thermoset matrix and an E-glass fabric. In situ polymerization was combined with liquid composite molding (LCM) techniques both in a resin transfer molding (RTM) mold and a lite-RTM (L-RTM) setup between two glass plates. The RTM specimens were used for mechanical characterization while the L-RTM samples were used for transmittance measurements. Optimization in terms of the number of glass fabric layers, the overall degree of transparency of the composite, and the mechanical properties was carried out and allowed for the realization of high mechanical strength and high-transparency tGFRPs. An outstanding degree of infiltration was achieved maintaining up to 75% transmittance even when using 29 layers of E-glass fabric, corresponding to 50 v.% fiber, using an L-RTM setup. RTM specimens with 44 v.% fiber yielded a tensile strength of 435.2 ± 17.6 MPa, and an E-Modulus of 24.3 ± 0.7 GPa.

## 1. Introduction

Fiber reinforcement in polymer composites provides high tensile strength, especially in a certain direction, and allows for the engineering of materials according to their application and loads needed, while keeping them lightweight. Also, due to the flexible structure of the textiles, the strength, stiffness, and impact resistance can be adjusted accordingly. As a result of these potentials, fiber-reinforced polymer composites are used especially in the aerospace, automotive, transport, and construction sectors, though with different requirements in terms of costs and quality. 

Optically transparent fiber-reinforced composites provide transparency as an additional function while maintaining the outstanding mechanical properties and lightweight character of the fiber-reinforced composites. Transparent composites have been used in a variety of applications including windshields with high-impact resistance [1] and lightweight armor [1]. Furthermore, applications in the areas of optics and magnetics [2,3,4], UV-protection [5], fire retardancy [6], and antibacterial equipment [7] have been reported. 

**Figure 1:**
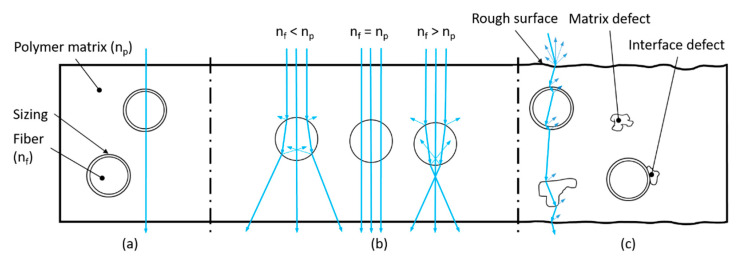
Optical effects of an incident ray permeating a tGFRP with (**a**) ideal properties: matching RI, smooth surface and no defects, (**b**) different levels of RI mismatch between polymer (n_p_) and fiber (n_f_) [14], and (**c**) realistic properties including defects that decrease the optical quality.

To obtain transparent tGFRPs, the loss in light transmittance must be minimized to the maximum possible extent. The following requirements need to be fulfilled to achieve this (Figure 1): (1) the refractive index matching between fiber and the polymer matrix, (2) the high infiltration quality of the resin system into the porous structure of the fiber reinforcement, and (3) the surface smoothness of the final composite.

The refractive index (RI) of the reinforcing fibers (n_f_) needs to be sufficiently close to the one of the matrix (polymer, n_p_) over the visible light spectrum and operating temperature range of the composite since any mismatch in RI would lead to chromatic aberration [8]. This is one of the major problems that impedes a high level of transparency in fiber-reinforced composites since both the polymer matrix and the fibers have differently changing RIs over the entire visible spectrum and temperature range. Generally, an increase in temperature decreases the RI of polymers [9,10]. Furthermore, even small deviations >0.001 in RI result in loss of transparency [8]. In view of these requirements, RI engineering of polymers [11] is a complex topic. The loss of transmission due to the deviations in RI between matrix and fiber is amplified with increasing fiber volume fraction. With a fiber volume fraction of 60 v.%, which is the established standard for highly stressed structural FRP parts [12], this is a challenging issue. 

The infiltration quality is the key to maintaining the optical properties of a tGFRP. Typical manufacturing induced issues such as matrix defects (incomplete polymerization, voids, contamination with binder agents or other process auxiliaries), fiber defects (fiber orientation deviation, waviness, fiber breakage, and irregular fiber distribution), interface defects (areas without bonding between matrix and fiber/sizing or fiber/textile layers—delamination, cracks) and surface defects (fiber print-through due to matrix shrinkage, irregular distribution of release agent residues, high or irregular distributed surface roughness, etc.) contribute to reduced transparency and must therefore be strictly avoided [8]. Furthermore, for ensuring proper wetting of the fibers, they have to be subjected to surface functionalization or must be equipped with a sizing, which also serves as a coupling agent to the matrix and also protects them during processing. The nature of the formed fiber-matrix interface not only influences the mechanical properties of the composite but also the light transmittance and optical clarity of it [13].

Finally, the surface of the tGFRP should have the highest possible smoothness, since any roughness in the sub-micron range results in light scattering and thus in lower transparency.

Several production methodologies for the manufacturing of different types of transparent fiber-reinforced composites exist. They differ in the selection of reinforcing fiber, textile structure, and matrix polymer type as well as manufacturing methods.

Epoxy-based resins are one of the widely applied systems as polymer matrices of tGFRPs [1,8,15,16,17,18,19,20,21,22,23]. Transparent composites made of epoxy-based resin systems [15,16] combined with a commercial-grade biaxially woven E-glass fabric with a vacuum-assisted resin transfer molding (VARTM) process were reported to have a tensile strength of 374.9 MPa, a tensile modulus of 31.74 GPa at a fiber volume fraction of 40.3 v.%. Jang et al. [17] manufactured transparent composites by casting various textile impregnations onto chopped fibers with different lengths, all made from E-glass fibers, and an epoxy-functionalized siloxane hybrid resin (EPSH), whose RI was tailored to that of the fibers. Zobeiry et al. [8] produced transparent composites from E-glass fabric and epoxy and polyester resins using a customized infusion process resulting in a composite with 45% fiber volume fraction and a transmittance of 78 to 88%, respectively. Glass fibers with different diameters (18, 37, 50 µm) were used to manufacture transparent composites between 25 and 45 v.% fiber fraction by casting an epoxy resin into an open tray, containing the unidirectional fiber reinforcement [18]. Transmittance values of 50–60% were achieved for the 45 fiber v.% specimens and wavelengths between 600–1100 nm. Ahmed and Khanna [24] fabricated transparent E-glass short fiber mat-reinforced, unsaturated polyester-matrix composites with a fiber volume of 15% using a sequential resin casting/fiber mat laminating process [25] and tested these specimens for their fracture strength and extreme testing temperatures. S-glass was used for enhanced mechanical properties. Krug et al. [19] made S-glass mat-reinforced epoxy composites using commercial 0/90 woven fiber mats while tailoring the RI of the matrix to match the fibers. To match the RI of the components, the epoxy resin was mixed with epoxy-functionalized polyhedral oligomeric silsesquioxanes (POSS). Blue and yellow chromatic dispersion, resulting from a slight RI mismatch between the fibers and the matrix, was observed. Meinders et al. [20] manufactured transparent composites panels ca. 1 mm in thickness from S-glass woven fabric with epoxy matrix using an adapted version of the VARTM process. The epoxy matrix was synthesized to match the RI of the fibers. They characterized the tensile, flexural, and impact properties of the composite. The transparency was visually inspected. Transparent composites were also prepared from glass ribbons to investigate the circular cross-section effect of the commercially available fabrics made of glass fibers [1,21,22,23]. Velez et al. [1,22] investigated epoxy resins, matched their RI with a soda-lime glass ribbon, and referred to the resulting composite as optically transparent ribbon-composite (OTRC). They achieved a glass volume fraction between 47 and 51 v.%, with transmittance values between 86.5 and 89.8% on OTRCs with a thickness between 1.9 and 2.9 mm.

Optically transparent glass-fiber-reinforced poly(methyl methacrylate) (PMMA) composites were developed [26,27] by matching the RIs of melt-spun BK10 glass fibers (13 µm in diameter, functionalized with a silane coupling agent) with the one of the PMMA matrix. The composite was manufactured by the casting of the monomer mixture into a closed mold, containing unidirectionally aligned fibers, followed by degassing and pressure curing. Self-reinforced poly(ethylene terephthalate) (PET) composites were reported to have a translucent structure with an elevated impact toughness due to anisotropy coming from the PET fibers [28]. 

The optical and tensile properties, material selection for fiber-reinforced composites based on either unidirectional continuous fibers or fabrics are summarized in Table S1 in the Supplementary Materials. 

Besides textile-based polymer composites, transparent polymer nanocomposites are also accessible by using nanoparticles and nanofibers [29,30,31,32,33,34,35,36,37,38,39,40,41,42,43]. In these composites, transparency can be achieved by the use of particles or fibers that are smaller than the wavelength of the visible light and thus scatter visible light less strongly [29]. A critical issue there is the agglomeration of these particles [30]. 

Here, we aimed for the production of tGFRP panels using commercial grade fiber reinforcement, resin systems, and manufacturing methods, which in the future could be upscaled to serial production. Therefore, commercially available resin components with a low mixing viscosity and plain woven E-glass fabrics, resulting in a good infiltration quality and optical transparency, were used. The RI of the resulting thermoset polymer was matched to the E-glass fabric. Two LCM manufacturing methods, i.e., RTM and L-RTM, respectively, were used to manufacture translucent and transparent specimens. Vacuum infusion-based L-RTM allowed for the manufacturing of composites with a smooth surface, thereby providing optical transparency. However, due to limited flow length, samples large enough for mechanical testing could not be produced with high fiber volume content. On the other hand, the RTM setup allowed for the additional use of injection pressure and enabled the manufacturing of larger samples though it did not provide the surface quality required for an optically tGFRP if the cavity surface of the RTM mold was not designed accordingly. 

## 2. Materials and Methods

### 2.1. Materials

**Figure 2:**
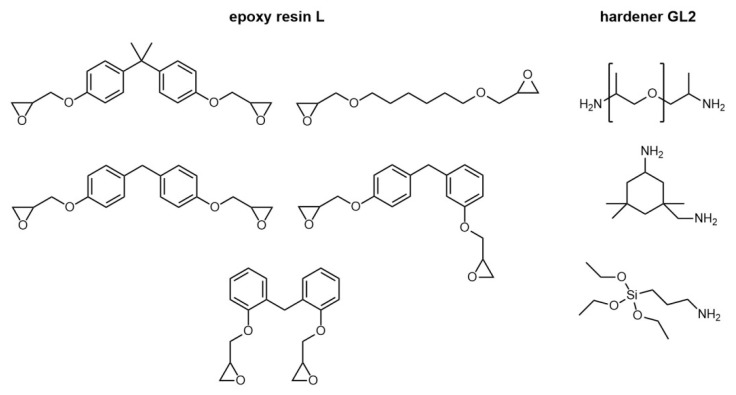
Chemical structures of the epoxy resin L (**left**) [45], and the hardener GL2 (**right**) [46].

Epoxy resin “L” and the hardener GL2 were purchased from R&G Faserverbundwerkstoffe GmbH (Waldenbuch, Germany). The chemical structures of the individual components are shown in Figure 2. The reaction mechanism of the epoxy-based systems is well established [44].

The mixing viscosity of the resin hardener mixture (100:30 wt.%) at 20 °C was 248 mPa·s [47] with a processing time of 210 min. Curing was accomplished within 24 h at room temperature, followed by 15 h at 60 °C [47]. E-Glass fabric (HexForce—02116 1260 TF970) [48] was purchased from Wela Handelsgesellschaft mbH, Germany. It was a plain-woven fabric with an areal weight of 106 g/m² (51 wt.% in the warp, 49 wt.% in the weft direction) from 22 tex yarns made of EC7 glass fibers with a density of 2.59 g/cm³. It was coated with an aminosilane finish (TF970). The materials were combined with tGFRP specimens with different fiber v.% using 5, 10, and 29 layers of textiles. The theoretical fiber volume fractions and corresponding layer numbers are shown in Table 1. The target thickness of the tGFRP specimens was 2 mm. 

### 2.2. Polymer Synthesis

The thermoset polymer matrix was synthesized inside the RTM and L-RTM molds, respectively. To prevent void formation, a satisfactory degree of degassing had to be accomplished. Prior to the mixing of the resin and hardener, degassing of these liquids was done individually in a vacuum desiccator for 1 h at 0.07 mbar. Subsequently, resin and hardener were dosed into a metal container in a 100:30 wt.% ratio and manually mixed using a wooden spatula. The mixed resin system was again degassed for 30 min at 0.07 mbar, since the mixing of these viscous liquids causes the entrapment of air.

### 2.3. Refractive Index (RI) Measurements

The RI of the resin and hardener were measured with the aid of an Abbe Mark III refractometer (Reichert Analytical Instruments, Buffalo, NY, USA) at 20 °C and 589 nm ($n_{D}^{20}$). The RI of the glass fabric was evaluated using RI liquids from the Cargille Laboratories (Cedar Grove, NY, USA) and an optical microscope (Zeiss AG, Oberkochen, Germany). 

### 2.4. Manufacturing of tGFRPs

#### 2.4.1. Manufacturing of Transparent Specimens with L-RTM

**Figure 3:**
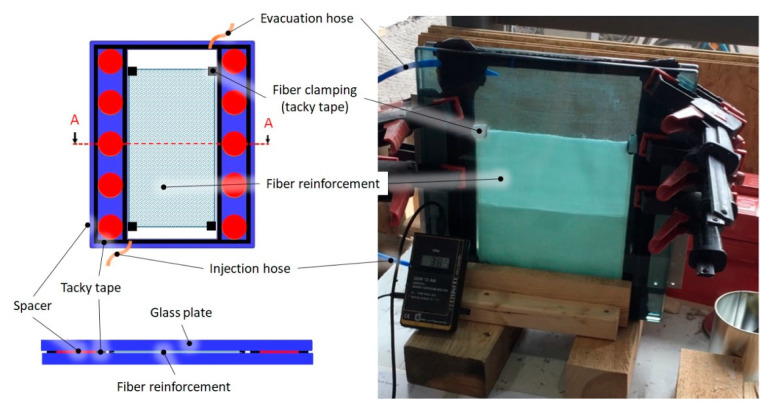
The L-RTM setup for manufacturing of the transparent specimens (tGFRP).

The L-RTM mold consisted of two rigid glass plates, separated by spacers and sealed with tacky tape SLT150B (Composyst GmbH, Landsberg am Lech, Germany) [49] (Figure 3).

**Figure 4:**
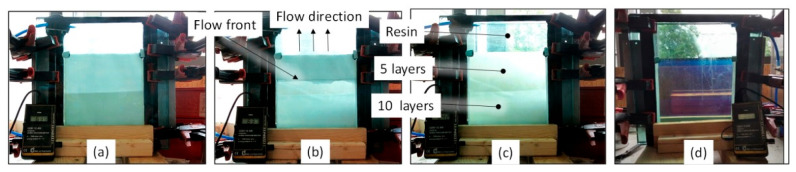
The L-RTM vacuum infusion states on the example of a preform (**a**) pre-infusion (**b**) during infusion (**c**) after infusion and set for cure (**d**) after 24 h of curing, with resin only (up), 5 layers (middle) and 10 layers (below).

The glass plates were cleaned and coated with a release agent, followed by polishing with a cloth. For cleaning, the acetone substitute Kieserol KF11 (Kiesewetter GmbH, Baiersdorf, Germany) and the water-based release agent Zyvax TakeOff (ChemTrend GmbH, Maisach, Germany) was used. For production of the tGFRP samples, the L-RTM setup was prepared as shown in Figure 3 and Figure 4a and evacuated to 12 mbar absolute pressure for 2 h. Figure 4 shows different processing states during infusion and curing. 

The resin and hardener components were prepared as described in Section 2.2. Before the resin was introduced into the L-RTM setup, the cavity pressure was raised to the start level of the infiltration pressure (Table 2). This pressure level correlated with the initial velocity of the flow front. Different initial pressure levels were used for different fiber volume fractions.

The injection hose was placed in the metal container with the degassed resin system and secured such that it was submerged below the resin level for infusion. Then, the injection hose was opened, the resin was slowly filled into the fiber-free volume below the reinforcement and allowed to progress as a linear flow front upwards (Figure 4b). 

The flow through the fiber reinforcement was manually controlled with a clamp on the inlet hose so that both the macro and microscopic flow front visually propagated at the same speed. After the cavity was filled, the inlet hose was clamped. For curing, the cavity pressure was raised to ambient pressure, which was around 940 mbar, and then sealed by clamping the evacuation hose. A significant change in the RI of the matrix during polymerization [50] together with clear differences in the optical transparency was observed (Figure 4c,d). Finally, the tGFRP specimen was demolded after an initial cure of 24 h at room temperature. Afterward, the specimen was post-cured at 60 °C for 15 h.

The manufactured L-RTM specimens were used for the characterization of the optical properties. It was not possible to manufacture samples large enough for mechanical characterization (350 × 230 mm²) with high fiber volume fraction (>10 layers), since controlling the flow front for a sufficiently long distance was difficult. Hence, another manufacturing method (RTM) was used for manufacturing specimens for characterization of the mechanical properties. 

#### 2.4.2. Manufacturing of Translucent Specimens with RTM

**Figure 5:**
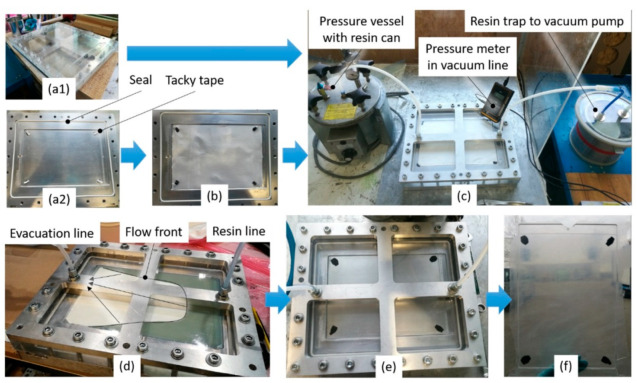
The RTM setup and its processing states: (**a1**) upper PMMA-mold, (**a2**) lower aluminum mold, (**b**) reinforcement in the cavity, (**c**) assembled mold, (**d**) resin injection, after 24 h of (**e**) cure, and (**f**) the demolded part.

The RTM process was used for manufacturing translucent specimens for material characterization and allowed for the production of larger samples (350 × 230 mm²) with fiber volume fractions of 0–60 v.%. The RTM mold consisted of two rigid mold sides, which have a seal in the parting plane and bolts for clamping the mold parts together (Figure 5a–c). The lower mold was made of aluminum, the upper of PMMA. The surfaces of both mold sides had a higher surface roughness compared to the L-RTM glass plates.

The RTM setup as well as the processing states are presented in Figure 5. The RTM manufacturing process started with the cleaning of the mold. The lower mold was treated according to the preparation of the L-RTM glass plates, while the PMMA mold was cleaned with isopropanol and coated with two different release agents. First, a layer of T7 release wax (ebalta Kunststoff GmbH, Rothenburg o.d.T, Germany), which was polished until the PMMA was transparent again, was applied. Second, a layer of PVA release film was applied on top of the release wax. About 30 min after application of the release agents the fiber reinforcement was placed inside the cavity of the lower mold. For low fiber volume fractions, tacky tape was used for positioning the fibers. Then the mold was closed, secured with bolts, and the injection, as well as the evacuation hose, were installed. The injections hose was connected to a pressure vessel, which later contained the resin container for infusion. The evacuation hose was connected to a resin trap and a vacuum pump.

The resin system was prepared according to the L-RTM trials. Pre-evacuation of the RTM setup was not possible, since no valves were available between the inlet point and the pressure vessel. Hence, the setup was ventilated to ambient pressure when the resin container was placed inside the pressure vessel. The infiltration pressure level was set while the inlet hose was not yet immersed below the resin level inside the resin container. As soon as the infiltration pressure was set according to Table 2, the hose was immersed into the resin and vacuum infiltration started immediately. Due to runners on the fiber reinforcement sides, the flow front did not propagate linearly but was rather U-shaped as presented in Figure 5d. To avoid the reduction in flow velocity that occurs with high fiber volume fraction so that capillary forced dominates viscous infiltration, the flow front was manually controlled to match capillary and viscous flow velocity by adjusting the injection pressure on the pressure pot. As soon as the mold was filled, the evacuation line was clamped and the injection pressure was raised to 5 bar for curing. The resin system was cured for 24 h at room temperature. After demolding, the part was post-cured at 60 °C for 15 h. 

### 2.5. Transmittance Intensity Measurement 

Transmittance data were obtained by placing the tGFRP samples in a Cary 60 UV-Vis Spectrophotometer (Agilent Technologies Germany GmbH & Co. KG, Waldbronn, Germany), after baseline and total absorption signal correction. At least two different places of the sample were measured to verify homogeneity.

### 2.6. Thermogravimetric Analysis (TGA)

TGA was done on a TGA Q500 V20.13 (Waters Corporation, New Castle, DE, USA) under N_2_ applying a heating rate of 10 °C/min from 30 °C to 900 °C. 

### 2.7. Differential Scanning Calorimetry (DSC)

DSC analysis was performed on a DSC Q2000 V24.10 (Waters Corporation, New Castle, DE, USA) applying a heating rate of 10 °C/min up to 150 °C.

### 2.8. Microscopic Analyses

For the microscopic analyses of the cross-sections of the specimens, they were first embedded in an epoxy resin (Struers EpoFix Resin from Struers GmbH, Willich, Germany). The grinding and polishing steps were accomplished on the Struers TegraPol-11 machine (Struers GmbH, Willich, Germany). Wet grinding (water-based) was done with diamond suspensions (Struers DiaPro 3 µm and 9 µm) while polishing was done with alumina suspensions (Buehler MasterPrep 0.05 µm from ITW Test & Measurement GmbH, Esslingen am Neckar, Germany) on different adhesive discs (Struers MD-Plan and Struers MD-Dur for diamond-grinding and Buehler Master Tex MD Rondo for alumina-polishing). While ZEISS Axioskop with DIC (differential interference contrast) was used for optical reflected-light-microscopy, a ZEISS Auriga field-emission SEM was the scanning electron microscope used in this study. For the preparation of SEM samples, the specimens were sputtered with a 5 nm thick layer of Pt-Pd (with Sputter Coater Quorum Technologies Q150T-ES from Quorum Technologies Limited, Laughton, East Sussex, UK).

### 2.9. Mechanical Testing

The tensile properties of the tGFRP as well as the pure polymer matrix were evaluated according to DIN EN ISO 527-4 (Typ 3) using the Hegewald & Peschke Universalprüfmaschine Inspekt 250 kN (Hegewald & Peschke Meß- und Prüftechnik GmbH, Am Gründchen, Germany) testing machine. The sample dimension was 250 × 25 mm² with a thickness of 2–3.5 mm. Each specimen type was tested at least seven times. The mechanical test results were obtained from the samples produced in the RTM setup.

## 3. Results

### 3.1. Infiltration Quality Data

**Figure 6:**
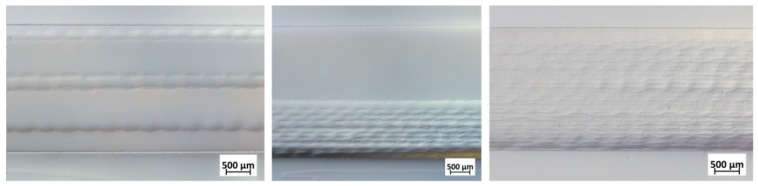
Light microscope images showing the cross-section of the composite samples manufactured via RTM with 5 (**left**), 10 (**middle**), and 29 layers (**right**) of E-glass fabric in a thermoset polymer matrix.

**Figure 7:**
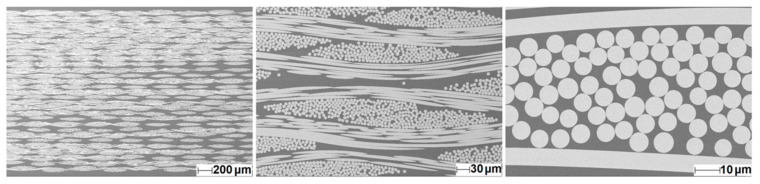
SEM images showing the cross-section of the 29 layers composite in different magnifications to evaluate the infiltration quality of the resin.

The resin infiltration quality by RTM setup and the final state of fabric distribution was evaluated from optical microscopy (Figure 6) and SEM analysis (Figure 7). While RTM samples containing 5 or 10 layers of fabric had a more heterogeneous distribution of the fabrics inside the matrix, a homogeneous distribution of the glass fabrics was obtained for the 29-layer RTM sample (Figure 6). 

With the 29 layers of E-glass fabric, the resin fully infiltrated the capillaries between the fibers, and each filament of the fabric was surrounded by resin (Figure 7). 

### 3.2. Transmittance Results

**Figure 8:**
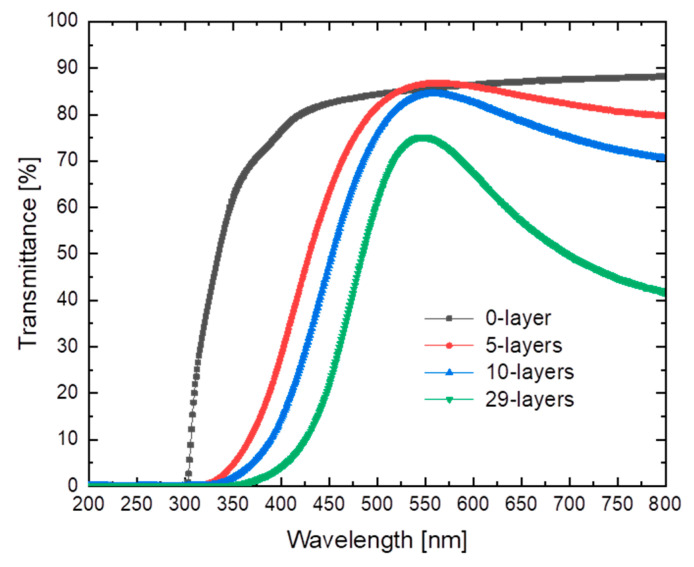
% Transmittance vs. wavelength for samples prepared by L-RTM containing 0, 5, 10, and 29 layers of glass fabric, respectively.

**Figure 9:**
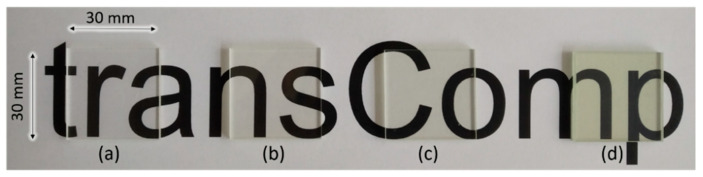
The naked-eye observation of the L-RTM specimens containing 0 (**a**), 5 (**b**), 10 (**c**), and 29 (**d**) layers of E-glass fabric.

The RI of the pure epoxy resin and the hardener was measured individually. The $n_{D}^{20}$ of Epoxy resin L was measured as 1.5527 ± 0.0001 while the hardener GL2 had an $n_{D}^{20}$ value of 1.4685 ± 0.0001. The best estimation for the RI of the E-glass fabric using the RI liquids was 1.5560 ± 0.0002 ($n_{D}^{25})$. The % transmittance values of the L-RTM samples are shown in Figure 8. The highest transmittance values having an onset at 300 nm and a constant transmittance ranging from 85 to 90% in the visible range (400–800 nm) were obtained for fabric-free samples. With an increasing number of layers of glass fiber (5, 10, 29), the onset of absorption was shifted towards 400 nm, attributable to an increased absorption or scattering of the fiber material, with a prominent transmittance maximum around 550 nm. The maximum transmittance values for the 5-, 10- and 29-layer samples were 87%, 85%, and 75%, respectively. For the 5- and 10-layer samples, the transmittance was almost the same as for the fabric-free reference sample. Towards the red and NIR (700–1000 nm), a slight decrease in transmittance down to ~70% was observed for the 5- and 10-layer samples, while transmittance decreased to 45% for the 29-layer sample. The characteristic transmittance at 589 nm and 20 °C was very high for all samples (Table 3), i.e., around 86% and 83%, for the 5- and 10-layer samples, and 70% for the 29 layer sample. Figure 9 illustrates the overall optical transparency of the L-RTM samples.

### 3.3. Mechanical Analysis

**Figure 10:**
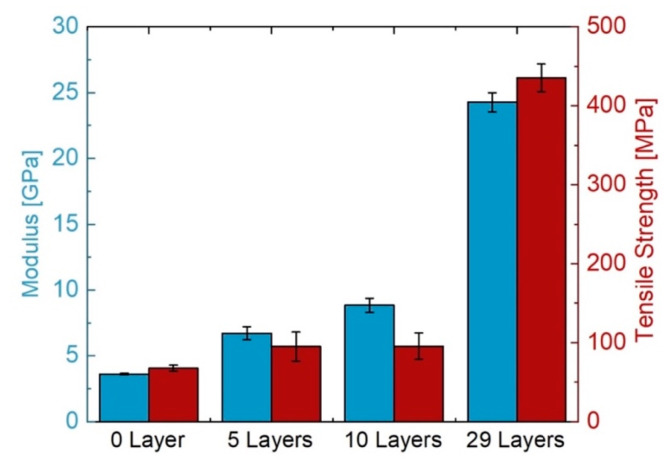
Mechanical properties of transparent composites in comparison to the unfilled resin (0 Layer).

Figure 10 depicts the mechanical properties of the composites and allows for a comparison with the pure polymer. Samples that were used for mechanical testing were produced via the RTM setup since this method allows for the production of homogeneous, void-free, and reproducible samples in the required dimensions for mechanical testing. Results are listed in Table 3. 

### 3.4. Thermal Characterization

Thermal characteristics of the tGFRP specimens and polymer matrix were evaluated by TGA (Figures S1 and S2, Supplementary Material) and DSC (Figure S3, Supplementary Material). The results are presented in the Supplementary Materials. 

## 4. Discussion

LCM manufacturing methods are suitable for the production of tGFRPs on a lab scale using commercially available and affordable materials. One of the challenges here was to avoid the use of any non-reacting or plasticizing chemicals in addition to the matrix system to match the RIs of the components. This led to good mechanical properties (Figure 10) while achieving the goal of good optical transparency (Figure 9).

For evaluation of the mechanical properties of the composites, the RTM technique had to be used, since manufacturing of larger size samples with high fiber volume fraction was not possible with the L-RTM setup. The RTM samples were translucent rather than transparent. Composites containing 29 layers of glass fabric, which had the highest fiber v.% of this study, showed outstanding infiltration quality (Figure 7) as a result of the low viscosity of the reaction mixture and a comparably slow reaction rate, which provides reasonable time for degassing and completing resin infusion before gelation starts. With this approach, there was enough time to control the micro- and macroscopic flow to minimize porosity. In fact, no porosity could be detected in the RTM samples at all (Figure 7). Due to this, the mechanical properties of the composites, summarized in Table 3, compete with those found in the literature (Table S1, Supplementary Material). Furthermore, DSC data showed that the reinforcing fibers did not affect the degree of curing, while TGA data presents a 10 °C increase in the onset decomposition temperature of the 29-layer composite compared to the neat polymer matrix. 

Composites containing 10-layers of glass fabric prepared by RTM were somewhat thicker, i.e., 3.4 mm compared to the target value of 2 mm, due to a varying pressure in the system during the curing phase. Nevertheless, the sample was still acceptable in terms of porosity despite its lower fiber v.% content (theoretically 20%, actually 12%). This sample also demonstrates how critical fiber distribution along the cross-section of the composite is. As can be seen in Figure 6, all of the 10-layers remained at the bottom of the sample while almost half of its cross-section remained unreinforced. This issue explains the almost identical tensile strength data of the 5- and 10-layer specimens. In case a micro-crack occurs at the fiber-free side of the testing specimen, it can easily propagate along the non-reinforced region, which induces a notch effect on the fiber-reinforced side, resulting in a stress concentration leading to the failure of the sample.

Generally, the production of composites using the L-RTM resulted in high transparency, being comparable to the reference sample for the 5- and 0-layer samples over large parts of the visible spectrum, even though the infiltration quality was not maintainable for the entire sample size. Additionally, the hand-made setup requires too much work, both in setup and detachment of the final composite for industrial applications. In conclusion, the L-RTM setup was accomplished to present the success of optical transparency. However, the technique itself is not yet applicable at an industrial scale because flow length at the high fiber volume fraction is limited due to the vacuum propelled infusion, which does not allow injecting the resin with pressure.

The L-RTM version of the 29 layers sample reached 50 v.% of fiber reinforcement while losing only 19% of transmittance compared to the pure polymer matrix at 589 nm. Thus, the transmittance value of the 29 layers containing the samples was 70% at standard 589 nm. However, up to 75% transmittance was observed for this sample at 549 nm, which corresponds to only 13% loss in transparency compared to the transmittance of the unfilled polymer at the same wavelength. It should be noted that the 10-layered L-RTM sample had a loss in transmittance of only 3.5% while the 5-layered sample had no detectable optical transmittance loss at 589 nm compared to the neat sample. 

As can be deduced from Figure 9, all composites are optically transparent. With an increasing number of textile layers, some minor changes in color occurred, which stemmed from a loss in transmittance in certain wavelength intervals (Figure 8), either caused by inevitable chromatic aberrations due to differences in RI between the glass fiber and the polymer over the visible wavelength spectra [8] or by light absorption in some parts of the visible spectrum, induced by the fibers embedded in the polymer matrix. Table 3 summarizes the transmittance and mechanical properties of selected specimens.

## 5. Conclusions

The production of optically transparent glass-fiber-reinforced composites based on a thermoset resin using both vacuum-assisted resin infiltration (L-RTM) and resin transfer molding (RTM) was successfully accomplished. The composites have been characterized in terms of infiltration quality, degree of transparency, mechanical and thermal properties. A good match in the RIs, smooth composite surfaces, and high infiltration quality have been achieved. The key to success was the low viscosity of the resin-hardener mixture. The good surface quality was accomplished via polymerization in a glass cavity of the L-RTM setup. The mechanical properties of the composites containing 5- or 10-layers of the glass fabric correlate with a heterogeneous distribution of these fabrics. By contrast, composites containing 29-layers, corresponding to 44 v.% of fiber, possess strongly enhanced mechanical properties. 

By matching the RIs of the materials at 589 nm, almost unchanged optical properties were obtained in this wavelength region for the 5- and 10-layer samples. Furthermore, compared to 86% of the pure polymer matrix, up to 75% transmittance was accomplished with the composite containing 29 layers of fabric, both prepared by L-RTM. A tensile strength of 435 MPa and a modulus of 24.3 GPa were achieved for the same composite, compared to 67 MPa strength and 3.6 GPa modulus of the polymer matrix, both prepared by RTM. Manual process control of the presented LCM manufacturing methods is challenging, particularly with regard to controlling sample thickness i.e., fiber v.%. Also, the flow front propagation requires better mold design, resin volume flow, and injection pressure control. 

For a homogeneous distribution of the textiles within the cavity, a new mold design combining the good surface quality of the L-RTM and the capability of the RTM setups to produce large-sized parts is required. Considering that commercially available resin systems and textiles were used in this study, the major limitation of the technology outlined here is related to upscaling and equipment. To satisfy these needs, a new RTM mold design and development is required that can provide an industry-scale, low porosity, and smooth surface production. 

## Figures and Tables

**Table 1:** Theoretical fiber v.% for selection of the number of textile layers.

Number of Fabric Layers	5	10	29
Theoretical fiber v.% for a composite thickness of 2 mm	10%	20%	60%

**Table 2:** Initial gas pressure (evacuation) inside the cavity at the beginning of the resin infusion.

Specimen	Resin Only	5 Layers	10 Layers	29 Layers
Initial cavity pressure (mbar)	850	640	640	50

**Table 3:** Summary of the measured optical and mechanical properties of the unfilled polymer and composites containing 5, 10, and 29 layers of glass fiber fabric.

Sample Type	Transmittance Specimens (L-RTM)				Mechanical Specimens (RTM)			
	Thickness (mm)	Fiber (v.%)	Transmittance (%)	Maximum Transmittance (%, Figure 8)	Thickness (mm)	Fiber (v.%)	Tensile Strength (MPa)	E-Modulus (GPa)
0-Layer	1.8 ± 0.1	0	86	89	2.6 ± 0.2	0	67.6 ± 4.0	3.6 ± 0.1
5-Layers	2.2 ± 0.1	9	86	87	2.5 ± 0.1	7	95.0 ± 18.3	6.7 ± 0.5
10-Layers	2.2 ± 0.1	19	83	85	3.4 ± 0.1	12	95.7 ± 16.6	8.8 ± 0.5
29-Layers	2.4 ± 0.1	50	70	75	2.7 ± 0.1	44	435.2 ± 17.6	24.3 ± 0.7

## Data Availability

Data Sharing is not applicable.

## Supplementary Materials

The following are available online at www.mdpi.com/article/10.3390/ma14206087/s1, Figure S1: TGA of a 0-layer sample prepared by RTM, Figure S2: TGA of a 29-layer sample prepared by RTM, Figure S3: DSC results of the RTM samples, Table S1: Summary of the properties of selected fiber-reinforced composites from the literature.
