# Supplementary material for: Transparent Fiber-Reinforced Composites Based on a Thermoset Resin Using Liquid Composite Molding (LCM) Techniques
Source: Materials (Basel). 2021 Oct 14;14(20):6087. doi: 10.3390/ma14206087 (PMC8538785; doi:10.3390/ma14206087)
Supplement: Supplementary file 1 [file materials-14-06087-s001.zip › materials-1405015-supplementary.pdf]

materials MDPI

Supplementary Materials

Transparent Fiber-Reinforced Composites Based on a Thermoset Resin Using Liquid Composite Molding (LCM) Techniques

Yavuz Caydamli [1,2,†], Klaus Heudorfer [3,†], Jens Take [3], Filip Podjaski [4], Peter Middendorf [3,*] and Michael R. Buchmeiser [1,2,*]

[1] Institute of Polymer Chemistry (IPOC), University of Stuttgart, Pfaffenwaldring 55, D-70569 Stuttgart, Germany; yavuz.caydamli@ipoc.uni-stuttgart.de (Y.C.)
[2] German Institutes of Textile and Fiber Research (DITF), Körschtalstr. 26, D-73770 Denkendorf, Germany
[3] Institute of Aircraft Design (IFB), University of Stuttgart, Pfaffenwaldring 31, D-70569 Stuttgart, Germany; heudorfer@ifb.uni-stuttgart.de (K.H.); take@ifb.uni-stuttgart.de (J.T.)
[4] Nanochemistry Department, Max-Planck-Institute for Solid State Research, Heisenbergstr. 1, D-70569 Stuttgart, Germany; podjaski@fkf.mpg.de (F.P.)
* Correspondence: peter.middendorf@ifb.uni-stuttgart.de (P.M.); michael.buchmeiser@ipoc.uni-stuttgart.de (M.R.B.)
† The authors contributed equally.

Table S1. Summary of the properties of selected fiber-reinforced composites from the literature.

Matrix	Reinforcement	%Transmission	Sample Thickness [mm]	Fiber v.%	Tensile Strength [MPa]	Modulus [GPa]	Ref.
PMMA	Melt-spun BK10 optical glass fibers	81.7 (600 nm, 20°C)	N/A	10.4	5.9	N/A	[21]
		44.8 (600 nm, 20°C)	N/A	34.8	19.5	N/A	
Epoxy	E-glass fibers	N/A	≈ 1	40.28	374.9 ± 23.53	31.74 ± 1.89	[24-25]
Epoxy	S-glass Fabric	84 (600 nm)	0.65	37	N/A	N/A	[26]
		71 (600 nm)	1.1	32	N/A	N/A	
		N/A	0.99	34	278 ± 9	16.4 ± 1.2	
Synthesized epoxy	Commercial woven fabrics made from S-glass fibers	N/A	≈ 1	N/A	624.6 ± 32.8	17.86 ± 1.32	[27]
EPSH	Continuous E-glass fiber mat	≈80 (550 nm)	1	25 wt%	≈160	N/A	[30]
EPSH	E-glass woven fabric	≈78 (550 nm)	1	10 layers	≈150	N/A	
EPSH	Chopped E-glass strands dispensed in EPSH	≈76 (550 nm)	1	25 wt%	≈65	N/A	
EPSH	Chopped strands + E-glass Woven fabric	80 (550 nm)	N/A	5 wt% + 10 layers	≈245	N/A	
Epoxy + MMA	Commercial woven fabrics made from E-glass fibers	88 (560 nm)	1	45	N/A	N/A	[8]
Polyester	Commercial woven fabrics made from E-glass fibers	78 (560 nm)	1	45	N/A	N/A	

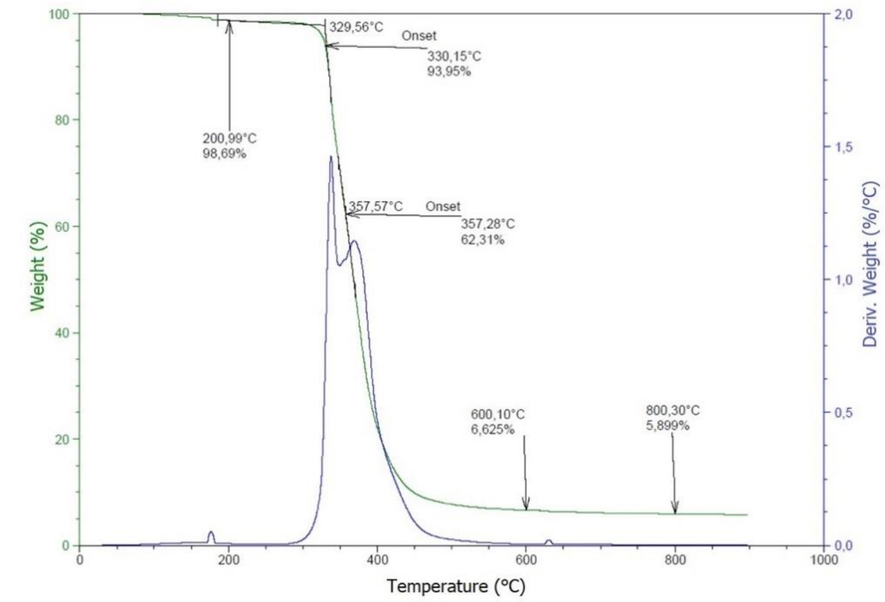

Figure S1. TGA of a 0-layer sample prepared by RTM.

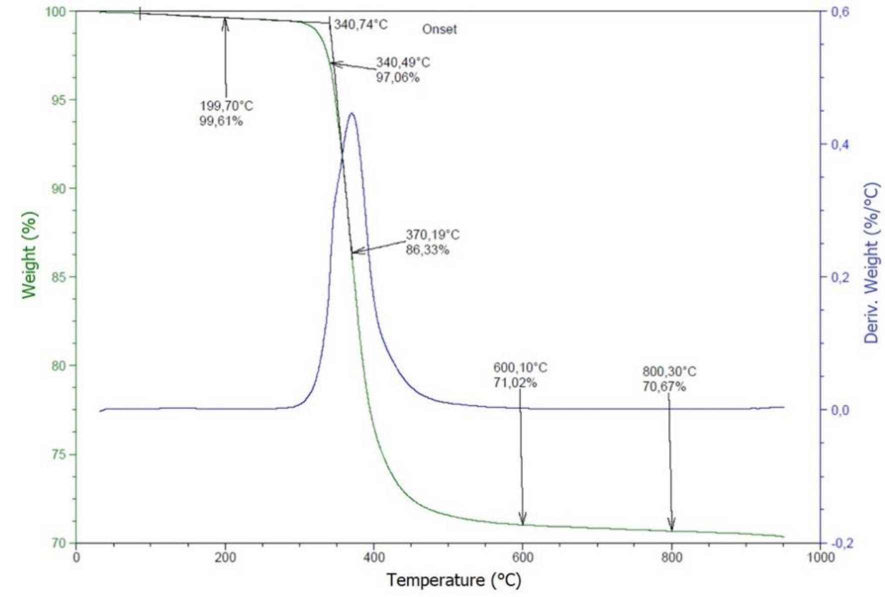

Figure S2. TGA of a 29-layer sample prepared by RTM.

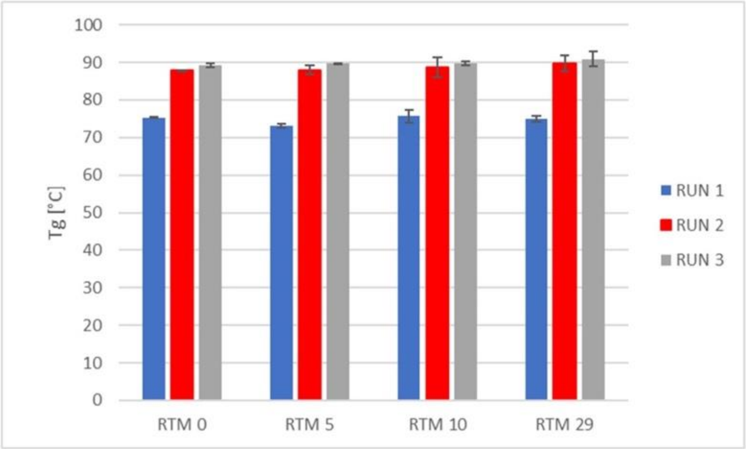

Figure S3. DSC results of the RTM samples.
